# Postoperative morbidity following surgery for mechanical adhesive small bowel obstruction: prevalence, risk factors and clinical outcomes

**DOI:** 10.1186/s12893-026-03670-0

**Published:** 2026-03-25

**Authors:** Timur Buniatov, Matthias Maak, Axel Denz, Christian Krautz, Georg F. Weber, Robert Grützmann, Anke Mittelstädt, Maximilian Brunner

**Affiliations:** Department of General and Visceral Surgery, Universitätsklinikum Erlangen, Friedrich-Alexander-Universität Erlangen-Nürnberg (FAU), Krankenhausstrasse 12, Erlangen, 91054 Germany

**Keywords:** Small Bowel Obstruction, Postoperative Complications, Morbidity, Surgical Outcomes, ASA physical status, Risk Factors, Adhesiolysis, Bowel Resection, Clavien-Dindo Classification

## Abstract

**Introduction:**

Despite its clinical significance and the fact that mechanical adhesive small bowel obstruction (SBO) is a common surgical emergency, data on the prevalence, types and risk factors of morbidity remain limited. This study aims to assess the frequency and kind of complications and identify patient- and surgery-related risk factors, with particular attention to the impact of surgical timing.

**Methods:**

We conducted a retrospective analysis of patients who underwent surgery for mechanical adhesive SBO at the University Hospital Erlangen over a five-year period (2018–2022). Postoperative complications and their outcomes were assessed. Univariate and multivariate logistic regression analyses were conducted to identify independent risk factors associated with postoperative morbidity.

**Results:**

Postoperative in-hospital morbidity occurred in 22.2% of patients, the majority of which were surgery-related (71.2%). The most frequent complications were postoperative paralytic ileus (6.0%), wound healing disorders (5.1%) and cardiopulmonary complications (5.1%). The in-hospital mortality rate was 2.1%. Postoperative morbidity was managed conservatively in 75.0% of cases and required reoperation in 25.0%. In 89.4% of affected patients, morbidity was associated with prolonged hospitalization. Multivariate analysis identified BMI > 25 kg/m² (OR 2.9; 95% CI, 1.4–6.0; p = 0.007), ASA class III–IV (OR 3.0; 95% CI, 1.4–6.5; p = 0.007) and surgery duration > 110 minutes (OR 4.0; 95% CI, 1.9–8.7; p < 0.001) as independent predictors of morbidity. In contrast, surgical timing – specifically nighttime procedures (p = 0.200) and weekend surgery (p = 0.572) - was not significantly associated with increased risk.

**Conclusion:**

In surgically treated patients with adhesive small bowel obstruction, postoperative morbidity was independently associated with elevated BMI, ASA class III–IV, and prolonged operative duration. In contrast, nighttime and weekend surgery were not significantly associated with postoperative morbidity in this cohort. These findings underscore the importance of risk-adapted perioperative optimization and close interdisciplinary management, particularly in high-risk patients.

## Introduction

Small bowel obstruction (SBO) is a common and potentially life-threatening surgical emergency, accounting for approximately 15% to 20% of all acute abdominal presentations in general and visceral surgery [1]. In Western countries, the leading cause of SBO is postoperative adhesions, reported in up to 70% of cases [2]. Patients are particularly predisposed following colorectal or gynecological procedures in the pelvic region [3].

Two main treatment strategies exist for SBO: surgical and conservative management. In recent years, there has been an increasing tendency toward conservative therapy. However, the key clinical challenge remains accurately identifying patients who require surgical intervention. As a result, extensive research has been devoted to defining predictive factors that help distinguish between patients who will benefit from operative versus non-operative management [4–8].

This decision-making process is further complicated by the rising number of elderly patients, many of whom present with multiple comorbidities, a history of prior abdominal operations and obesity - factors that have repeatedly been shown to increase the risk of postoperative morbidity and mortality [9, 10]. Notably, more than 20% of patients presenting with SBO eventually undergo surgery [11]. These cases often reveal complex intra-abdominal findings such as distended, fragile bowel loops and dense adhesions, which increase the risk of intraoperative complications, particularly iatrogenic injuries.

A major concern in this context is bowel ischemia, which occurs in approximately 20% of adhesive SBOs, in up to 40% of closed-loop obstructions, and in as many as 60% of volvulus cases [12]. Once irreversible ischemia develops, bowel resection becomes necessary, further raising the risk of postoperative complications and mortality [9].

The occurrence of complications in SBO patients significantly influences clinical outcomes and presents considerable challenges for surgeons. Morbidity rates can reach 30%, with reoperation rates reported at up to 9% [13], but can rise to as high as 14.5% in specific subgroups, such as patients with malignant obstruction [14]. Beyond the clinical implications, the economic burden of SBO is substantial, with estimated annual healthcare costs in the United States alone approaching $1.3 billion [15].

Given this considerable clinical and economic impact, there is a strong need to better understand the role of patient-related factors, intraoperative complexity and surgical timing in determining outcomes. Despite this, a notable gap persists in the literature regarding the detailed characterization of complications in SBO patients and the identification of specific risk factors contributing to their occurrence.

Therefore, the aim of this study is to systematically assess the prevalence and types of postoperative complications in patients with SBO and to identify independent patient-related, intraoperative and timing-related risk factors associated with these adverse outcomes.

## Materials and methods

### Study design

This retrospective study included adult patients who were hospitalized and surgically treated for small bowel obstruction (SBO) at the University Hospital Erlangen (Germany) between 1 January 2018 and 31 December 2022. An initial screening identified 771 patients presenting with bowel obstruction of various etiologies and anatomical locations.

**Fig. 1:**
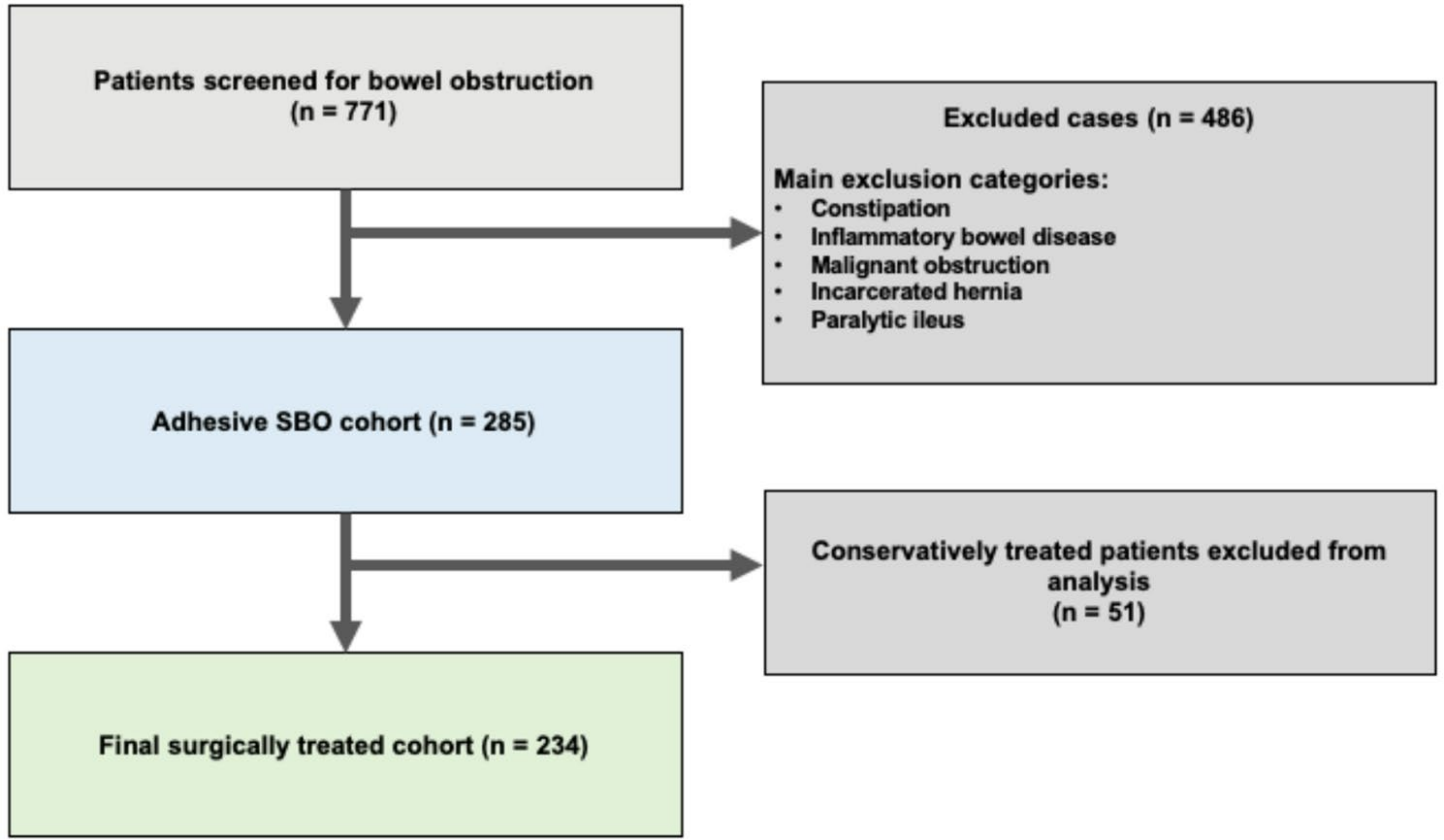
Patient selection and study cohort flow diagram.

To obtain a homogeneous study cohort focused specifically on mechanical adhesive SBO, the following exclusion criteria were applied: malignancy-related obstruction, colonic obstruction, paralytic ileus, subileus (defined as partial bowel obstruction without relevant laboratory abnormalities, with imaging findings indicating impaired bowel passage or incomplete obstruction, and managed without operative treatment), inflammatory bowel disease (Crohn’s disease or ulcerative colitis), incarcerated hernias, incomplete medical records, and other diagnoses inconsistent with mechanical adhesive SBO. These conditions differ substantially in pathophysiology, treatment strategy, postoperative risk profile, and clinical course and were therefore excluded from the present analysis. The final analysis included 234 surgically treated patients with mechanically confirmed adhesive SBO. A detailed description of patient selection and cohort formation is provided in the Results section and illustrated in Fig. 1.

Clinical data were retrospectively extracted from electronic hospital records, including demographic characteristics, clinical presentation, comorbidities, preoperative diagnostics such as laboratory and imaging findings, treatment variables and postoperative course.

Comorbidities were retrospectively extracted from patient medical records and included major pre-existing conditions relevant to perioperative risk, such as cardiovascular disease, diabetes mellitus, chronic obstructive pulmonary disease, and liver cirrhosis. Further subcategorization (e.g., coronary artery disease vs. heart failure; Type 1 vs. Type 2 diabetes) was not uniformly documented in the retrospective dataset and therefore could not be analyzed separately. Individual comorbidities were considered in the descriptive and univariate analyses. However, because these variables represent overlapping aspects of baseline systemic risk, and to avoid redundancy while preserving an adequate events-per-variable ratio in the multivariate model, ASA Physical Status was used as the primary summary measure of preoperative patient risk. ASA Physical Status scores [16] were obtained from the anesthesia records of all operated patients. For regression analysis, ASA was dichotomized into ASA I–II versus ASA III–IV to provide a more standardized and clinically generalizable representation of overall comorbidity burden, while also reducing model overfitting.

Postoperative complications were identified and categorized as surgical or nonsurgical, with further specification into one of eight subtypes: wound healing disorder, burst abdomen, postoperative paralytic ileus, anastomotic insufficiency, enterocutaneous fistula, cardiac complications, pulmonary complications and other internal medical complications. The severity of complications was graded according to the Clavien–Dindo classification [17]. Categorization into different Clavien–Dindo grades was performed by a single investigator (first author, T.B.) based on predefined objective criteria. In cases of uncertainty or complex clinical courses, the classification was discussed and confirmed in consensus with a senior surgeon (M.B.). Outcomes of complicated cases were documented and classified as conservative or operative treatment, with or without prolonged hospital stay. Prolonged length of stay (LOS) was defined as hospitalization beyond the 75th percentile (> 16 days) of all included patients.

Due to the retrospective design of the study and the anonymized use of patient data, the requirement for individual informed consent was waived. The study protocol, including this waiver, was reviewed and approved by the Ethics Committee of Friedrich-Alexander University Erlangen-Nürnberg (Approval Number: 24-414-Br) and was conducted in accordance with the principles of the Declaration of Helsinki.

### Definitions

Due to the emergency setting, only routinely available laboratory parameters at our university hospital were analyzed. Serum albumin was used as a surrogate marker for nutritional status and C-reactive protein (CRP) as an indicator of systemic inflammation. Other parameters such as prealbumin, procalcitonin (PCT), or nutritional risk scores (e.g., NRS 2002) were not consistently documented in emergency cases and were therefore not included in the analysis. Laboratory-based risk factors were defined using clinically relevant pathological thresholds. A serum creatinine level > 1.2 mg/dL was considered abnormal, based on the reference range of 0.7–1.1 mg/dL at our institution. C-reactive protein (CRP) was dichotomized at > 75 mg/L, reflecting a markedly elevated inflammatory response and in accordance with previous studies linking high preoperative or early postoperative CRP levels to increased morbidity in emergency abdominal surgery [18]. For serum albumin, the normal range at our institutional laboratory is 35–50 g/L. Hypoalbuminemia was defined as < 34 g/L, in line with values established in previous studies associating low albumin levels with malnutrition and adverse postoperative outcomes [19–21].

In the absence of a universally accepted age cut-off for this setting, age > 68 years was used as a pragmatic study-specific threshold. This corresponded to the median age of the overall SBO cohort analyzed in our related previous study and closely approximated the median age of the present surgically treated subgroup, thereby allowing consistent and clinically interpretable exploratory risk stratification [8]. BMI was dichotomized at > 25 kg/m² according to the standard WHO definition of overweight [22].

For operative duration, ROC curve analysis was performed using postoperative complications as the binary outcome. The optimal threshold according to the maximum Youden index was approximately 105 min and was operationalized as a clinically practical cut-off of > 110 min for dichotomized analysis and reporting.

The level of surgical experience was recorded as resident or expert. “Resident” referred to procedures performed by surgeons in postgraduate training, whereas “expert” referred to board-certified attending surgeons or consultants in the German surgical training system. Surgical timing was categorized as daytime (06:00–18:00) or nighttime (18:00–06:00), with the latter being performed by the night-shift surgical team. Surgeries were also classified as weekday or weekend/official holiday procedures, reflecting the reduced on-call staffing during non-working days. Additionally, a subanalysis specifically examined outcomes of operations performed during the late-night period between 00:00 and 06:00.

### Surgical approach and management of complications at our center

The diagnosis of mechanical small bowel obstruction in our cohort was established based on a combination of patient history, clinical presentation, laboratory findings, imaging results, and intraoperative confirmation, in accordance with current surgical and radiological standards. Radiological assessment included abdominal X-ray, ultrasonography, and computed tomography (CT) when indicated. Surgical decisions regarding the timing and type of operative intervention were made by experienced consultant surgeons. In all patients, standard preoperative management included nasogastric decompression in cases of vomiting and intravenous fluid resuscitation to correct dehydration and electrolyte imbalances. By contrast, patients who were initially managed conservatively and later required surgery could receive parenteral nutritional support as part of conservative treatment during bowel rest. Patients managed successfully without surgery were not included in the present analysis.

The choice between laparoscopic and open surgery was guided by anticipated complexity, particularly the extent of adhesions. Intraoperatively, complete adhesiolysis was performed with the aim of restoring bowel continuity. Intestinal viability was routinely assessed, and resection was indicated in cases of irreversible ischemia or perforation. Reconstruction was typically performed using a hand-sewn end-to-end anastomosis, employing either a continuous single-layer absorbable monofilament 4 − 0 suture or interrupted absorbable braided 3 − 0 sutures, with the technique left to the discretion of the operating surgeon based on intraoperative conditions.

Diagnosis of postoperative complications was based on clinical, laboratory, and imaging criteria according to standardized surgical practice. Postoperative paralytic ileus was defined by abdominal distension, nausea or vomiting, absence of stool or flatus for more than three postoperative days, and/or the need for nasogastric tube reinsertion. In patients with severe or unclear postoperative symptoms, computed tomography (CT) was routinely performed to identify or exclude intra-abdominal complications such as anastomotic leakage, abscess, or bowel injury.

Postoperative complications were managed according to standard protocols. Superficial wound infections were generally treated conservatively on the surgical ward, often allowing for discharge and outpatient care. Prolonged postoperative ileus was managed with nasogastric decompression, prokinetic agents and, when indicated, short-term parenteral nutritional support. Anastomotic leaks required re-laparotomy. Patients with cardiopulmonary deterioration were managed in the interdisciplinary intensive care unit (ICU) in close collaboration with the cardiology and pulmonology departments.

### Statistical analysis

All statistical analyses were performed using Jamovi Software version 2.3.28 (Sydney, Australia). Descriptive statistics were used to summarize the overall complication rate, its distribution across Clavien–Dindo grades, and the prevalence of specific complication types within the study cohort, as well as their associated outcomes.

Univariate analyses were conducted to assess potential associations between selected risk factors and the occurrence of postoperative complications. Continuous variables were analyzed using the Mann–Whitney U test, while categorical variables were compared using the chi-square (χ²) test, as appropriate. Variables with a p-value < 0.05 in univariate analysis were subsequently entered into a multivariate binary logistic regression model. For the multivariate analysis, only dichotomized categorical variables were included. A backward stepwise logistic regression procedure was applied to identify independent predictors of postoperative morbidity. Odds ratios (ORs) with corresponding 95% confidence intervals (CIs) were calculated for all variables retained in the final model.

To illustrate the relationships between main risk factors, complication profiles, subspecific complication types and clinical outcomes, a Sankey diagram was constructed. Main risk factors were defined according to statistically significant variables from the multivariate model. Each patient with complications was assigned a dominant risk factor, determined according to the highest odds ratio identified in the multivariate model. Complication profiles were classified as surgical or nonsurgical and further subdivided into predefined subspecific categories. Clinical outcomes were grouped into: (1) conservative therapy followed by normal discharge, (2) conservative therapy with prolonged length of stay (LOS), defined as hospitalization beyond the 75th percentile (> 16 days), (3) Reoperation combined with prolonged LOS, (4) in-hospital mortality. The width of each flow in the Sankey diagram reflected the number of patients within the respective pathway.

## Results

### Patient selection and baseline characteristics

Of the 771 patients initially screened for bowel obstruction, 285 remained after application of all exclusion criteria and formed the adhesive SBO cohort. Among the excluded patients, the most frequent categories were constipation/fecal impaction (156 patients, 20.2%), inflammatory bowel disease—mainly Crohn’s disease with chronic terminal ileal or anastomotic strictures (125 patients, 16.2%)—and partial bowel obstruction (subileus) managed conservatively (111 patients, 14.4%), often in patients with multiple previous abdominal operations or a known frozen abdomen. Less frequent exclusion categories included malignant obstruction (26 patients, 3.4%), other causes of bowel obstruction (23 patients, 3.0%), large-bowel obstruction (22 patients, 2.9%), incarcerated hernia (16 patients, 2.1%), and paralytic ileus (7 patients, 0.9%).

**Fig. 2:**
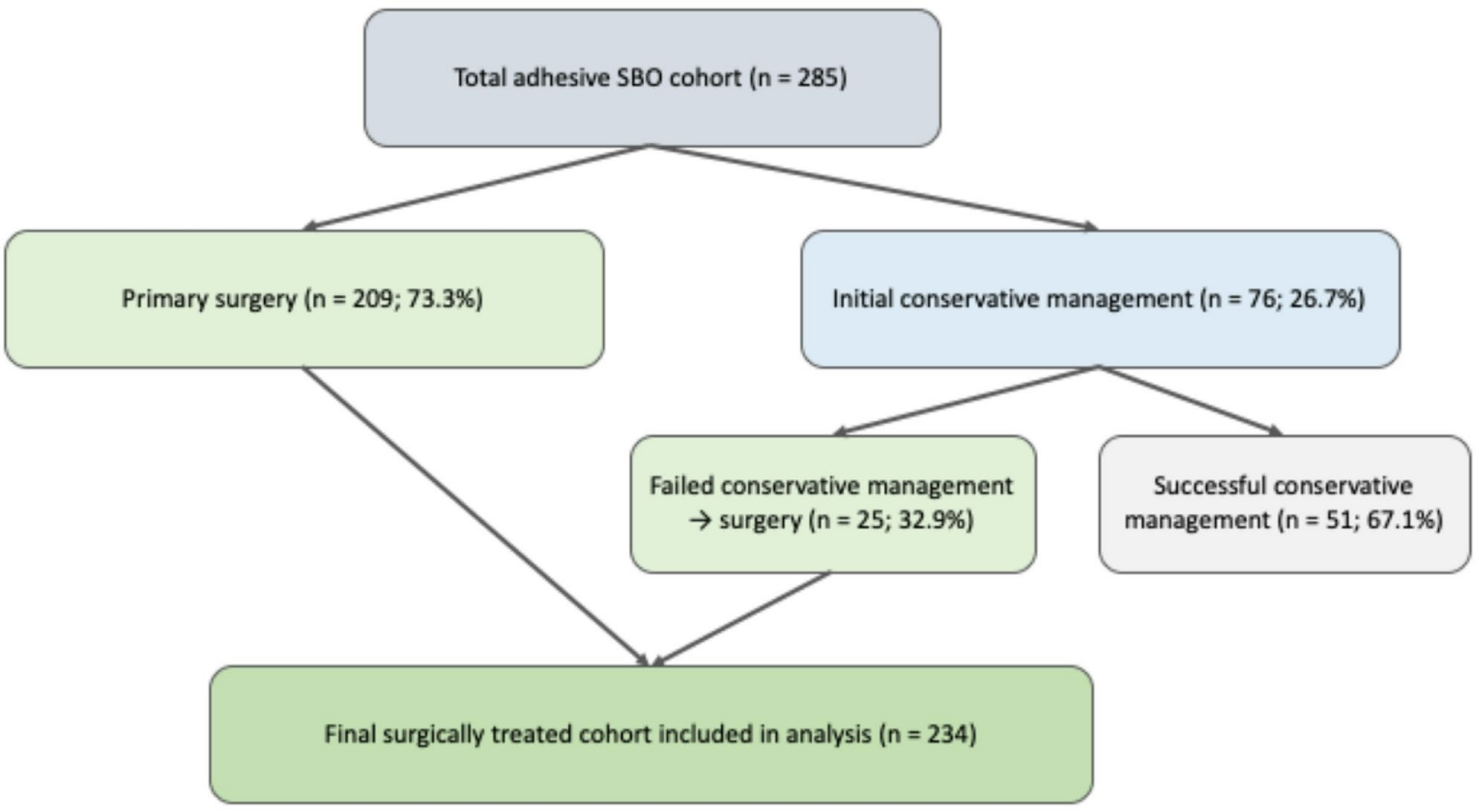
Management flow diagram of patients within the adhesive small bowel obstruction cohort. Percentages are calculated relative to the corresponding parent group

Within this adhesive SBO cohort, 209 patients (73.3%) underwent primary surgery at presentation, whereas 76 patients (26.7%) were initially managed conservatively. Among these 76 patients, 25 (32.9%) ultimately required surgery because of unsuccessful conservative treatment. In routine clinical practice, the duration of initial conservative management was usually around 1–3 days; however, this interval was not documented in a sufficiently standardized manner to allow formal analysis. Consequently, 234 patients with surgically confirmed mechanical adhesive SBO constituted the final study cohort. The patient selection process is illustrated in Fig. 1, and the management pathway is summarized in Fig. 2.

The decision to proceed from conservative treatment to surgery was made by an experienced consultant surgeon, often after case discussion within the surgical team. The main indications for surgery were clinical or laboratory deterioration, failure of obstruction to resolve under conservative management, and/or radiologic evidence of persistent mechanical obstruction, particularly on computed tomography. Baseline clinical characteristics of the final study cohort are summarized in Table 1. The most prevalent comorbidities in the final study cohort were cardiovascular disease in 53 patients (22.6%), diabetes mellitus in 28 patients (12.0%), chronic pulmonary disease in 20 patients (8.5%) and liver cirrhosis in 6 patients (2.6%).

**Table 1:** Preoperative and surgical risk factors associated with postoperative morbidity in surgically treated patients with adhesive small bowel obstruction

Variable		All Patients (*n* = 234)	Patients with complications (% or median [IQR]) (*n* = 52)	Patients without complications (% or median [IQR]) (*n* = 182)	*p*-value
Age (median [IQR])		67 (15)	76 (15.3)	64 (25)	**< 0.001**
Gender (n (%))	Male Female	112 (47.9) 122 (52.1)	30 (57.7) 22 (42.3)	82 (45.1) 100 (54.9)	0.108
BMI (kg/m^2^) (median [IQR])		25.1 (6.5)	28.8 (7.5)	24.2 (6.0)	**< 0.001**
ASA I (n (%)) ASA II (n (%)) ASA III (n (%)) ASA IV (n (%))		27 (11.5) 92 (39.3) 107 (45.7) 8 (3.4)	1 (1.9) 12 (23.1) 33 (63.5) 6 (11.5)	26 (14.3) 80 (44.0) 74 (40.7) 2 (1.1)	
ASA III–IV vs. ASA I–II (n (%))	III–IV I–II	115 (49.1) 119 (50.9)	39 (75.0) 13 (25.0)	76 (41.8) 106 (58.2)	**< 0.001**
Cardiovascular disease (n (%)) Diabetes (Type 1 and 2) (n (%)) COPD (n (%)) Liver cirrhosis (n (%))		53 (22.6) 28 (12.0) 20 (8.5) 6 (2.6)	22 (42.3) 15 (28.8) 12 (23.1) 3 (5.8)	31 (17.0) 13 (7.1) 8 (4.4) 3 (1.6)	**< 0.001** **< 0.001** **< 0.001** 0.097
Pain character (n (%))	Colicky Continuous	198 (84.6) 32 (13.7)	47 (90.4) 5 (9.6)	151 (83.0) 27 (14.8)	0.330
Preoperative CRP level (mg/L) (median [IQR])		12.2 (40.3)	21.4 (64.1)	10.3 (29.4)	**0.003**
Preoperative albumin level (g/L) (median [IQR])		32 (7.0)	29.5 (9.3)	33 (7.1)	**< 0.001**
Preoperative creatinine level (mg/dl) (median [IQR])		0.9 (0.47)	1.1 (0.52)	0.8 (0.4)	**< 0.001**
Air-fluid levels on abdominal X-ray (n (%)) (*n* = 187)*	Yes No	148 (79.1) 39 (20.9)	34 (87.2) 5 (12.8)	114 (77.0) 34 (23.0)	0.165
Free intraperitoneal fluid in imaging (Sonography or CT) (n (%))	Yes No	170 (72.6) 64 (27.4)	37 (71.2) 15 (28.8)	133 (73.1) 49 (26.9)	0.784
Transition point on CT (n (%)) (*n* = 226) *	Yes No	217 (96.0) 9 (4.0)	50 (100) 0 (0)	167 (94.9) 9 (5.1)	0.103
Duration of surgery (min) (median [IQR])		108 (46.2)	150 (85.0)	90 (77.5)	**< 0.001**
Surgeon experience (n (%))	resident specialist/consultant	30 (12.8) 204 (87.2)	7 (13.5) 45 (86.5)	23 (12.6) 159 (87.4)	0.875
Daytime (06:00–18:00) vs. nighttime (18:00–06:00) surgery (n (%))	Nighttime Daytime	168 (71.8) 66 (28.2)	41 (78.8) 11 (21.2)	127 (69.8) 55 (30.2)	0.200
Late-night surgery (00:00–06:00) (n (%))	Yes No	102 (43.6) 132 (56.4)	25 (48.1) 27 (51.9)	77 (42.3) 105 (57.7)	0.459
Weekend surgery (n (%))	Yes No	100 (42.7) 134 (57.3)	24 (46.2) 28 (53.8)	76 (41.8) 106 (58.2)	0.572
Small bowel resection (n (%))	Yes No	47 (20.1) 187 (79.9)	17 (32.7) 35 (67.3)	30 (16.5) 152 (83.5)	**0.010**

*ASA* American Society of Anesthesiologists Physical Status classification, *BMI* Body mass index, *CRP* C-reactive protein, *IQR* Interquartile range. Continuous variables are reported as median (IQR), and categorical variables as number of patients (n) and percentage (%). Percentages were calculated column-wise, representing the proportion within each subgroup (patients with complications and patients without complications)

* Missing data for the specified variable

With regard to the ASA physical status classification, only classes I–IV were observed. The most frequent category was ASA III, documented in 107 patients (45.7%). Overall, 115 patients (49.1%) were classified as ASA III–IV and 119 patients (50.9%) as ASA I–II.

### Operative timing, surgeon experience, and surgical approach

A substantial proportion of all SBO surgeries, (168 cases, 71.8%), were performed at night, with 102 surgeries (43.6%) occurring during the late-night period (00:00–06:00). In total, 100 cases (42.7%) were operated on during weekends or official holidays. Neither nighttime surgery nor late-night surgery showed a statistically significant association with the occurrence of complications (*p* = 0.200 and *p* = 0.459, respectively). Similarly, no correlation was found between surgeries performed on weekends or holidays and the development of complications (*p* = 0.572). Most SBO surgeries were performed by experienced surgeons (board-certified attendings or consultants), accounting for 204 cases (87.2%), while 30 cases (12.8%) were performed by residents. However, the surgeon’s level of experience did not significantly influence the complication rate in our cohort (*p* = 0.875). Detailed results are presented in Table 1.

Among the 234 patients included in the study, open surgery was performed in 211 cases (90.2%), whereas a laparoscopic or laparoscopically assisted approach was attempted in 23 cases (9.8%). Conversion to open surgery was required in 11 of these patients (47.8%), primarily due to extensive adhesions or impaired visualization secondary to bowel distension. The surgical approach was selected at the discretion of the operating surgeon based on the anticipated complexity of adhesiolysis, intraoperative findings and the patient’s prior surgical history.

### Observed complications, their classification, and associated outcomes

In the final cohort of 234 patients undergoing surgery for adhesive SBO, 52 patients (22.2%) developed postoperative morbidity. The distribution of complications according to the Clavien–Dindo classification, their profiles (surgical vs. nonsurgical), specific subtypes, and associated outcomes are summarized in Table 2.

**Table 2:** Postoperative morbidity and outcomes in patients undergoing surgery for adhesive SBO

		*n* (%)
Total study cohort		234 (100.0)
Postoperative morbidity	Yes No	52 (22.2) 182 (77.8)
Clavien-Dindo grade	0	182 (77.8)
	I	15 (6.4)
	II	9 (3.8)
	III	8 (3.4)
	IV	15 (6.4)
	V	5 (2.1)
Complication type (*n* = 52)	Wound healing disorder Postoperative paralytic ileus	12 (5.1) 14 (6.0)
	Burst abdomen	4 (1.7)
	Anastomotic insufficiency	5 (2.1)
	Enterocutaneous fistula	2 (0.9)
	Cardiac complications	3 (1.3)
	Pulmonary complications	9 (3.8)
	Other internal medical complications	3 (1.3)
Complication profile (*n* = 52)	Surgical Nonsurgical	37 (15.8) 15 (6.4)
Complication outcome (*n* = 52)	Conservative therapy + normal discharge Conservative therapy + Prolonged LOS Reoperation + Prolonged LOS Mortality	5 (2.1) 29 (12.4) 13 (5.6) 5 (2.1)

*LOS* Length of stay

According to the Clavien–Dindo classification, complications were distributed as follows: grade I, 15 patients (6.4%); grade II, 9 patients (3.8%); grade III, 8 patients (3.4%); grade IV, 15 patients (6.4%); and grade V, 5 patients (2.1%). When stratified by complication profile, surgical complications were more frequent than nonsurgical complications, occurring in 37 patients (15.8%) versus 15 patients (6.4%), respectively. The most common surgical complications included postoperative paralytic ileus (*n* = 14; 6.0%), wound-healing disorders (*n* = 12; 5.1%), anastomotic insufficiency (*n* = 5; 2.1%), burst abdomen (*n* = 4; 1.7%) and enterocutaneous fistula (*n* = 2; 0.9%). Among nonsurgical complications, pulmonary complications were the most common (*n* = 9; 3.8%), followed by cardiac complications (*n* = 3; 1.3%) and other internal medical complications such as acute kidney injury or sepsis (*n* = 3; 1.3%).

Regarding clinical outcomes in complicated cases (*n* = 52), the most frequent was conservative therapy with prolonged length of stay (LOS), observed in 29 patients (12.4%). This was followed by reoperation combined with prolonged LOS in 13 patients (5.6%). Normal discharge after conservative therapy occurred in 5 patients (2.1%), while in-hospital mortality was recorded in 5 patients (2.1%).

### Univariate analysis of risk factors for postoperative complications

Univariate analysis identified eight factors significantly associated with the development of postoperative complications: advanced age (> 68 years; *p* = 0.003), body mass index (BMI) > 25 kg/m² (*p* < 0.001), higher ASA class (ASA III–IV; *p* < 0.001), elevated preoperative C-reactive protein (CRP) > 75 mg/L (*p* = 0.039), preoperative hypoalbuminemia (serum albumin ≤ 34 g/L; *p* = 0.017), preoperative hypercreatininemia (≥ 1.2 mg/dL; *p* < 0.001), prolonged operative time (> 110 min; *p* < 0.001), and the need for small bowel resection (*p* = 0.010). In addition, several individual comorbidities were also associated with postoperative complications in univariate analysis, including cardiovascular disease (*p* < 0.001), diabetes mellitus (*p* < 0.001), and chronic obstructive pulmonary disease (COPD) (*p* < 0.001), whereas liver cirrhosis was not significantly associated (*p* = 0.097). Because individual comorbidities reflect overlapping aspects of baseline systemic risk, they were not entered separately into the multivariate model. Instead, ASA III–IV was used as the primary summary measure of preoperative patient risk.

### Multivariate logistic regression analysis

In the final multivariate binary logistic regression analysis, three independent risk factors for postoperative complications were identified: BMI > 25 kg/m² (OR 2.9; 95% CI 1.4–6.0; *p* = 0.007), ASA class III–IV (OR 3.0; 95% CI 1.4–6.5; *p* = 0.007), and an operative duration > 110 min (OR 4.0; 95% CI 1.9–8.7; *p* < 0.001) (Table 3).

**Table 3:** Univariate and multivariate analysis of risk factors for postoperative complications

Variable	Univariate	Multivariate		
	*p*-value	OR	95% CI	*p*-value
Age (> 68 vs. ≤68 years)	**0.003**	–	–	0.206
BMI (> 25 vs. ≤25 kg/m²)	**< 0.001**	2.9	1.4–6.0	**0.007**
ASA class III–IV vs. ASA I–II	**< 0.001**	3.0	1.4–6.5	**0.007**
Preoperative CRP level (> 75 mg/L vs. ≤75 mg/L)	**0.039**	–	–	0.629
Preoperative albumin level (≤ 34 g/L vs. >34 g/L)	**0.017**	–	–	0.125
Preoperative creatinine level (≥ 1.2 mg/dL vs. <1.2 mg/dL)	**< 0.001**	–	–	0.140
Duration of surgery (> 110 min vs. ≤110 min)	**< 0.001**	4.0	1.9–8.7	**< 0.001**
Small bowel resection (yes vs. no)	**0.010**	–	–	0.183

*OR* Odds ratio, *CI* Confidence interval, *CRP* C-reactive protein, *ASA* American Society of Anesthesiologists Physical Status classification. ORs and 95% CIs are shown for variables retained as independent predictors in the final multivariate model

### Correlation of risk factors with complication types and clinical outcomes

**Fig. 3:**
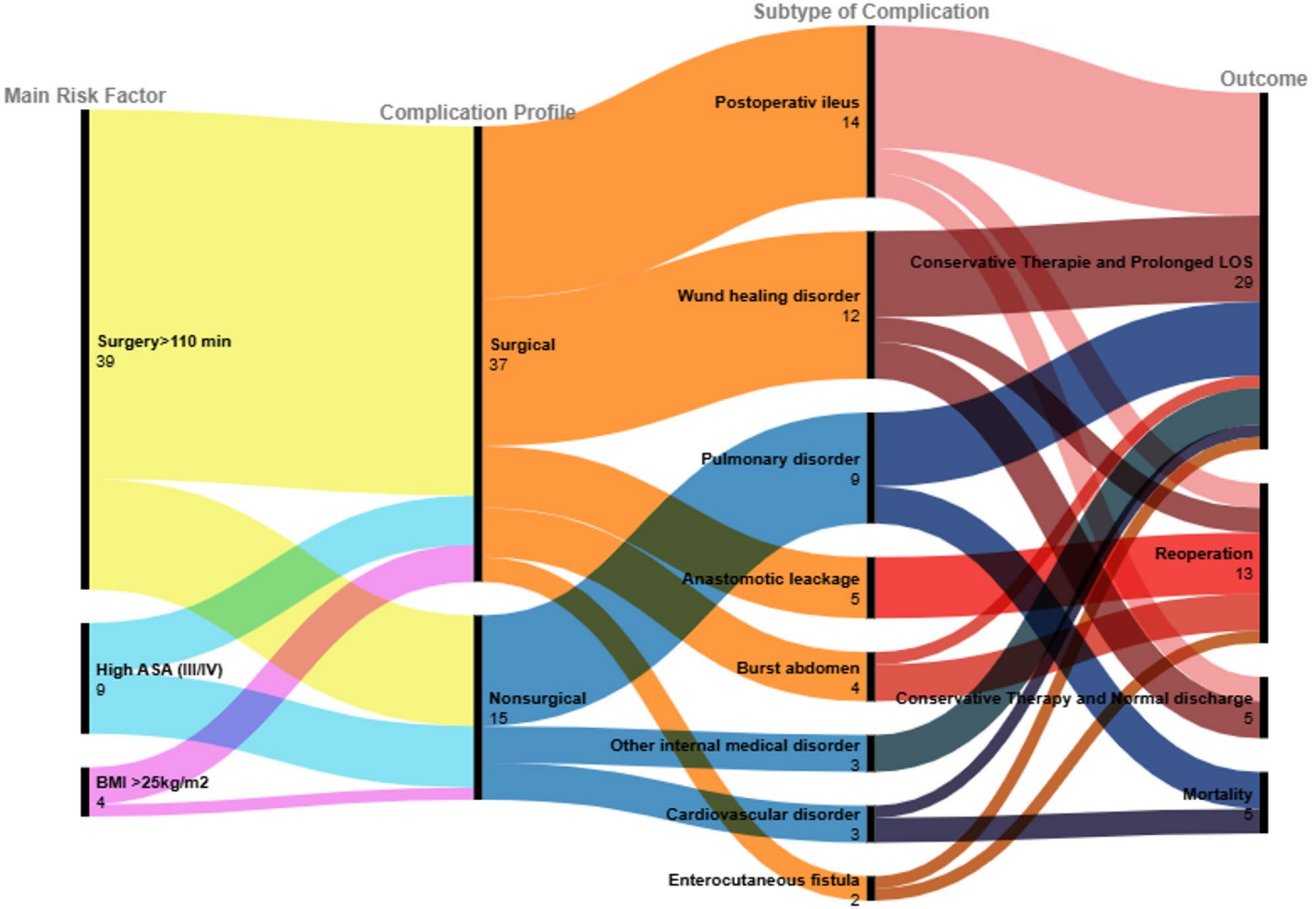
Sankey diagram of main risk factors, complication types and clinical outcomes. For visualization purposes, each patient with postoperative morbidity was assigned to one predominant risk factor only. This approach was used solely to simplify the Sankey diagram and does not fully capture the multifactorial nature of postoperative complications

The Sankey flow diagram illustrates the associations between major risk factors, specific complication profiles, and their corresponding clinical outcomes (Fig. 3). The visualization showed that prolonged operative duration (> 110 min) was most frequently associated with surgical complications, which in turn were more often linked to reoperation. In contrast, patients with higher baseline systemic risk (represented by ASA III–IV) were more likely to develop nonsurgical complications, commonly resulting in prolonged hospital stays and, in some cases, mortality. However, aside from the associations between surgical complications and reoperation (*p* = 0.008) and between nonsurgical complications and mortality (*p* < 0.001), no other relationships reached statistical significance.

## Discussion

Limited data exist regarding postoperative morbidity following surgical treatment for small bowel obstruction (SBO). This study provides a comprehensive analysis of postoperative complications and identifies key risk factors that may guide perioperative management and risk stratification.

In our cohort, 22.2% of patients experienced postoperative morbidity, consistent with published rates ranging from 15% to 30% for SBO surgery [23]. Complications included both surgical and non-surgical events, with a ratio of approximately 2:1. Surgical complications were primarily postoperative paralytical ileus and wound healing disorder, while non-surgical complications were mainly cardiopulmonary in nature. Notably, all fatal outcomes in our study were due to non-surgical complications. This suggests that the pre- and postoperative cardiopulmonary status of patients plays a more decisive role in postoperative mortality than the surgical trauma itself, which is typically moderate in cases of adhesiolysis with or without small bowel resection [24].

Multivariate analysis identified three independent risk factors significantly associated with increased postoperative morbidity:

First, elevated Body Mass Index (BMI) > 25 kg/m² was associated with a higher risk of morbidity. Obesity is a well-documented risk factor for surgical complications. It increases technical difficulty due to poor visualization, longer operative times and impaired wound healing [25]. Obese patients are also more prone to wound infections and incisional hernias, primarily due to compromised tissue perfusion and increased dead space [26]. Moreover, systemic effects of obesity - such as impaired respiratory function, increased thromboembolic risk and altered immune response - can adversely affect recovery [27]. The frequent coexistence of comorbidities like diabetes mellitus further compounds these risks. These findings highlight the importance of incorporating BMI into preoperative risk stratification and carefully weighing surgical indications in obese patients.

Second, pre-existing comorbidities such as cardiovascular disease, diabetes mellitus or chronic kidney disease have repeatedly been associated with an increased risk of postoperative complications in SBO patients [25]. In our cohort, this burden of comorbidity was reflected by higher ASA classes: ASA III–IV emerged as an independent predictor of postoperative complications after SBO surgery (OR 3.0, *p* = 0.007). This finding is in line with previous reports. In one study, ASA classes IV and V were identified as significant risk factors for morbidity in patients operated for SBO [9]. Jeppesen et al. likewise found that an ASA class of ≥ III was strongly associated with postoperative morbidity among patients undergoing emergency surgery for small bowel obstruction in univariate analysis [10]. In patients with closed-loop obstruction, an ASA class ≥ III has also been reported as a predictor of irreversible ischemia [28]. In a multicenter prospective cohort of patients operated on for adhesive small bowel obstruction, an ASA class of III or higher was associated with medical complications (OR 16.8), which in turn constituted an important risk factor for mortality [29]. Taken together, these data highlight the central role of global physiological reserve, as captured by the ASA classification, in determining outcomes after SBO surgery. Pre-existing comorbidities limit cardiopulmonary and metabolic reserves and impair the ability to tolerate surgical stress and postoperative complications [30]. Consequently, careful preoperative optimization, early involvement of anesthesiology and intensive care teams, and enhanced postoperative monitoring appear particularly important for high-risk patients with ASA III–IV and should be systematically considered in perioperative decision-making and resource allocation.

Third, prolonged operative time emerged as a significant independent risk factor of postoperative complications. Longer surgeries are often associated with increased complexity, difficult adhesiolysis or intraoperative complications. In our cohort, operations exceeding 110 min were independently associated with a higher risk of postoperative morbidity (*p* < 0.001, OR 4.0). This is consistent with a meta-analysis by Cheng et al., which showed that the risk of complications doubles when operative time exceeds two hours [31]. Lengthy operations are linked to risks such as hypothermia, coagulopathy, prolonged anesthesia exposure and greater tissue trauma. In particular, extended adhesiolysis increases the risk of inadvertent enterotomies, potentially leading to leaks or fistulas [32]. The need for small bowel resection further reflects the intraoperative complexity, as patients requiring resection exhibited higher morbidity compared with those undergoing adhesiolysis alone (*p* = 0.010), consistent with prior studies [9]. The morphology and extent of adhesions are key determinants of operative difficulty. Differentiating between simple membranous adhesions and dense, matted, or vascularized adhesions—as well as distinguishing single bands from diffuse inter-enteric involvement—can greatly improve risk assessment [33, 34].

Several validated scoring systems, such as the Zühlke Classification (grades 0–4) [35] and the Peritoneal Adhesion Index (PAI) [36], provide structured approaches to quantify adhesion severity and anatomical distribution. Higher PAI scores have been associated with an increased risk of re-obstruction and re-intervention [37]. Unfortunately, these classifications are rarely used in emergency settings due to time constraints, lack of standardization in operative documentation, and limited awareness among surgeons. Consequently, adhesiolysis difficulty was not assessed in our cohort.

Several additional variables showed significant associations in univariate analysis but did not reach statistical significance in multivariate analysis: hypoalbuminemia, a surrogate marker of malnutrition and systemic inflammation, was significantly associated with increased morbidity in univariate analysis. Although this association did not persist in the multivariate model, existing literature supports its clinical relevance [19, 24–27, 38, 39]. Malnutrition is known to impair wound healing and immune defense, increasing susceptibility to infections, anastomotic dehiscence and sepsis [40, 41].

An emerging body of literature suggests that prealbumin, owing to its shorter half-life, is a more sensitive indicator of acute nutritional changes than albumin [42]. However, its predictive value in mechanical SBO remains uncertain, and it is not routinely available in emergency settings. Studies in gastrointestinal and oncologic surgery have demonstrated that lower prealbumin levels are associated with wound infections, higher postoperative morbidity, and poorer overall outcomes [40, 43, 44]. Further investigations are required to determine its prognostic utility specifically in SBO patients.

Given that univariate analysis identified hypoalbuminemia as a significant predictor, the use of composite nutritional assessment tools could provide a more comprehensive evaluation of perioperative risk. Indices such as the Nutritional Risk Index (NRI) [45], Prognostic Nutritional Index (PNI) [46], and Nutritional Risk Screening 2002 (NRS-2002) [47] combine biochemical and anthropometric parameters (e.g., albumin concentration, body weight changes, and total lymphocyte count), thereby capturing the complex interaction between malnutrition, inflammation, and immune function.

For example, the PNI—a composite marker reflecting both nutritional and inflammatory status—has shown strong prognostic value in predicting postoperative morbidity and survival. In a study by Lee et al., SBO patients with low NRI values had a 4.2-fold higher in-hospital mortality, while Ge et al. reported that higher PNI scores correlated with reduced postoperative morbidity. Similarly, elevated NRS-2002 scores were linked to prolonged nil-per-os duration and extended hospital stay, underlining their clinical utility for standardized risk stratification in emergency surgery [47–49].

In addition to nutritional parameters, C-reactive protein (CRP) remains a well-established inflammatory marker. While CRP lost statistical significance in our multivariate model, previous studies consistently identified CRP > 75 mg/L as predictive for severe SBO and the need for surgical intervention [18, 50–52]. Given that albumin is a negative acute-phase protein, the CRP-to-albumin ratio (CAR) has recently gained attention as an integrated prognostic marker reflecting both inflammation and nutritional depletion [53]. Future studies should further explore CAR in the context of SBO, as preliminary evidence suggests its potential superiority in predicting adverse outcomes.

Another promising biomarker is procalcitonin (PCT), which demonstrates higher specificity than CRP for detecting bacterial translocation, intestinal ischemia, and early postoperative sepsis [54–56]. Although PCT was not assessed in our dataset, its inclusion in future SBO studies may substantially enhance early risk stratification and perioperative decision-making in acute settings.

A particular focus of our investigation was the effect of operative timing and surgical experience on outcomes. The so-called “weekend effect” - worse outcomes during off-hours - has been previously discussed, but remains controversial in SBO surgery [57]. In our cohort, neither nighttime nor weekend/holiday surgeries were significantly associated with higher complication rates (*p* = 0.200 and *p* = 0.572, respectively). This aligns with findings from McVay et al. and Butensky et al. [58, 59]. The high proportion of nighttime operations (71.8%) in our cohort was influenced by institutional logistics. Because daytime operating room capacity was limited and often prioritized for complex elective oncological procedures, urgent SBO surgeries were frequently performed during evening or nighttime hours. Although nighttime and weekend surgery were analyzed, the actual interval from diagnosis to surgery was not assessed in the present study. This may represent an additional relevant determinant of postoperative outcome and should be addressed in future prospective investigations.

Regarding surgeon experience, we found no significant difference in complication rates based on the experience level of the operating surgeon. However, given that 87.2% of procedures in our cohort were performed by experienced surgeons, the study may have been underpowered to detect such differences. To date, the literature provides limited evidence on the direct impact of surgeon experience on outcomes in SBO surgery [60].

The identification of these risk factors may have practical implications for perioperative care. High-risk patients may benefit from aggressive perioperative optimization and intensified postoperative monitoring. Our findings support the development of risk stratification tools to aid surgical decision-making and resource allocation.

Strengths of our study include the detailed categorization of complication profiles, comprehensive outcome tracking and statistically robust risk factor modeling. However, several limitations should be acknowledged. First, the retrospective single-center design and the moderately sized cohort limit the generalizability of our findings. Institutional differences in surgical strategy, postoperative management, and patient selection may influence complication rates and outcomes, thereby affecting the external validity of the results. In addition, only surgically treated patients with adhesive SBO were included, while conservatively managed cases were not assessed. Therefore, our findings apply specifically to the surgically managed subgroup and should not be extrapolated to the entire population of patients with adhesive SBO. Furthermore, the actual interval from diagnosis to surgery (time-to-surgery) was not assessed in the present analysis. Although nighttime and weekend surgery were evaluated, these variables do not fully capture treatment delay or disease progression prior to operation. Time-to-surgery may represent an additional determinant of postoperative morbidity and should therefore be addressed in future prospective studies.

Second, as a tertiary university hospital, our institution frequently treats more complex cases, including referrals from smaller regional hospitals that lack intensive care capacity or specialized surgical expertise. Such patients often present with advanced disease or after failed conservative management, leading to a higher baseline risk and potentially worse outcomes. On the other hand, the consistently high surgical standards, availability of specialized teams, and comprehensive postoperative intensive care at a university hospital setting may have mitigated the adverse effects typically associated with emergency timing or case complexity. These institutional characteristics may therefore explain both the relatively high overall morbidity and the relatively low mortality rate observed in our cohort.

Third, only in-hospital outcomes were evaluated, as reliable data on 30- or 90-day readmission and mortality were not consistently available in the retrospective dataset. Future prospective multicenter studies are needed to assess longer-term outcomes and to further validate the present findings.

## Conclusion

In surgically treated patients with adhesive small bowel obstruction, elevated BMI, ASA class III–IV, and prolonged operative duration were independent predictors of postoperative morbidity. These findings support careful preoperative risk stratification and tailored perioperative management, particularly in high-risk patients.

## Data Availability

The datasets generated and analyzed during the current study are not publicly available due to the presence of personal information related to patients. However, they are available from the corresponding author upon reasonable request.

## Abbreviations

SBO: Small bowel obstruction
CRP: C-reactive protein
LOS: Length of stay
AKI: Acute kidney injury
BMI: Body mass index
ASA: American Society of Anesthesiologists Physical Status classification
CT: Computed tomography
COPD: Chronic obstructive pulmonary disease
ROC: Receiver operating characteristic
